# Exercise Motivation and Strength Training among Older Adults with and without a Cancer History

**DOI:** 10.1093/geroni/igaf122.2738

**Published:** 2025-12-31

**Authors:** Nancy Gell, Myeongjin Bae

**Affiliations:** University of Vermont, Burlington, Vermont, United States; University of Vermont, Burlington, Vermont, United States

## Abstract

Strength training is beneficial for addressing cancer-related and age-related muscle loss. We aimed to 1) describe strength training among US older adults by cancer history and 2) examine motivating factors for exercise and their association with strength training. Nationally representative data of adults age ≥65 years from the Health Information National Trends Survey (HINTS) from 2012 and 2019 were analyzed (n = 2854). Participants reported days of strength training/week and how much four motivating factors (appearance, enjoyment, guilt, pressure from others) reflect why they start or continue exercising regularly. Analyses included descriptive statistics and Poisson logistic regression; analyses were weighted to account for complex sampling design. Overall, 35% of older adults reported participation in any strength training. Among older adults with a cancer history, 22.8% reported strength training 2+ days/week versus 29.9% of those without a cancer history (p = 0.02). Appearance was associated with meeting strength training recommendations among older adults without a cancer history (Incidence rate ratio [IRR] 1.6, 95% confidence interval [CI]: 1.3-1.9) but not among those with a cancer history. Enjoyment as a motivating factor was significantly associated with strength training among both groups (IRR: 2.3, 95% CI: 1.9-2.9, overall) whereas pressure from others was not associated with meeting strength training recommendations in either group. Less than half of U.S. older adults participate in strength training with significantly lower participation among older adults with a cancer history. Enjoyment is a strong motivating factor associated with meeting strength training recommendations and a potential target for exercise promotion efforts.

